# Clinical Isolation and Identification of *Haematospirillum jordaniae*

**DOI:** 10.3201/eid2410.180548

**Published:** 2018-10

**Authors:** Gregory Hovan, Andrew Hollinger

**Affiliations:** Delaware Public Health Laboratory, Smyrna, Delaware, USA (G. Hovan);; Regional Hospital, Delaware^1^ (A. Hollinger)

**Keywords:** Delaware, Haematospirillum jordaniae, H. jordaniae, novel pathogen, bacteria, United States

## Abstract

A clinical case study involving a man (35–49 years of age) with wounds to his lower right extremity. An isolate was sent to the Delaware Public Health Laboratory for confirmatory testing by genetic analysis of the 16S gene. Testing identified the isolate as a novel genus and species, *Haematospirillum jordaniae*.

The Centers for Disease Control and Prevention (CDC) recently recognized a human pathogen, *Haematospirillum jordaniae*, as a new bacterial genus and species (*1*). *H. jordaniae* is a gram-negative, spiral-shaped aerobe in the family *Rhodospirillaceae* (*1*). Before the pathogen’s identification, this organism was isolated 14 times from 10 different states. These organisms were received by CDC’s Special Bacteriology Reference Laboratory, Division of High-Consequence Pathogens and Pathology, National Center for Emerging and Zoonotic Infectious Diseases, over the course of 10 years (February 2003–October 2012) before the initial publication. Here we report the clinical identification of *H. jordaniae*, a potential emerging infectious pathogen, by the Delaware Public Health Laboratory (DPHL) and describe possible routes of infection.

A man (35–49 years of age) sought care at a hospital emergency department in September 2016 with worsening pain, redness, and swelling of the right lower extremity, which had an open wound on the right shin. The patient stated that he had finished a project cutting stones 3 weeks earlier where the water and stone chips were hitting his right leg. The patient ignored swelling, erythema, and fever he experienced around the same period when the stonecutting occurred and decided not to seek medical intervention until he was no longer able to ignore the symptoms.

The patient was found to have cellulitis of the right leg and sepsis, and he was admitted to the hospital for treatment. On the same day, he was started on intravenous clindamycin, and blood cultures were collected. The infectious disease physician discontinued clindamycin and placed the patient on vancomycin, ciprofloxacin, and aztreonam because of concern for potential infection with *Pseudomonas*, *Aeromonas*, or methicillin-resistant *Staphylococcus aureus*. Growth was observed in both sets of aerobic bottles, the first after 2 days, 15 hours, and the second after 4 days, 18 hours. Blood cultures grew gram-negative bacilli, leading the infectious disease physician to remove vancomycin but continue with ciprofloxacin and aztreonam. The patient improved before identification of the pathogen and was discharged on oral ciprofloxacin.

Gram stain of the organism revealed small, curved, gram-negative bacilli, resembling *Campylobacter*. Subcultures from the bottles did not grow aerobically until 48 hours after incubation on sheep’s blood agar and chocolate agar. The organisms did not grow on MacConkey agar or when incubated anaerobically on blood agar. Although it was suspected that the organism might be a *Campylobacter* species, it did not grow in a microaerophilic environment. Biochemical testing revealed the organism as oxidase-positive, indole-negative, catalase-positive, and urease-negative (using urea agar). The isolate was sent to the DPHL for identification.

DPHL received a grown isolate from the submitting hospital 8 days after the patient’s admission. The organism was cultured in the same manner as described previously; phenotypic tests were consistent with the findings of the submitting hospital. Initial identification was performed at DPHL, but the pathogen could not be determined because the organism was not part of the instrument’s stored database.

The isolate’s DNA was extracted and amplified by using general methods described in numerous publications. The sample generated an 845-bp portion of the 16S region. The assembled sequence was uploaded to open-source rRNA databases for comparison, including GenBank BLAST (https://blast.ncbi.nlm.nih.gov/Blast.cgi), the Ribosomal Database Project (https://rdp.cme.msu.edu/), and MicrobeNet (https://microbenet.cdc.gov). Samples most closely matched to *Novispirillum itersonii* strain LMG_4337^T^ (92.3% identity). On the basis of Clinical Laboratory Standards Institute guidelines (*2*), this similarity indicated a possible novel species.

The DPHL Microbiology Department’s laboratory manager sent the organism to CDC’s Special Bacteriology Reference Laboratory. Results identified the organism as *H. jordaniae*. Upon review of MicrobeNet at a later date, the organism was found to match *H. jordaniae* H5569_con^T^ by 98.9%.

*H. jordaniae* is a common environmental microbe, but it was implicated in this clinical case in a man in Delaware. The patient had symptoms characteristic of other pathogenic bacterial illnesses. Concern exists that slow-growing, gram-negative rods identified in blood culture could be potential bioterrorism agents. Humrighouse et al. (*1*) described how *Francisella tularensis* infection was suspected in 2 clinical cases that were actually *H. jordaniae* infections.

Humrighouse et al. (*1*) proposed the name *H. jordaniae* on the basis of an isolate received in 2010. Previously, 14 organisms identified at CDC were isolated from blood taken from men 39–78 years of age with symptoms including swelling of the lower extremities (2 patients), septicemia (3 patients), and bacteremia (1 patient). The symptoms of the patient we report mirrored those symptoms.

This discovery is important because it demonstrates that organisms conceived to be environmental in nature and suspected to have limited clinical implications are emerging as human pathogens. The ability to identify bacteria by sequencing (in this case, sequencing of the 16S rRNA gene) was necessary to identify *H. jordaniae* because clinical information on this pathogen is still limited.

## References

[1] Humrighouse BW, Emery BD, Kelly AJ, Metcalfe MG, Mbizo J, McQuiston JR. *Haematospirillum jordaniae* gen. nov., sp. nov., isolated from human blood samples. Antonie van Leeuwenhoek. 2016;109:493–500. 10.1007/s10482-016-0654-0 26857139

[2] Clinical and Laboratory Standards Institute. Interpretive criteria for identification of bacteria and fungi by DNA target sequencing. Approved guideline (MM18-A). Wayne (PA): The Institute; 2008.

